# Visualising inter-subject variability in fMRI using threshold-weighted overlap maps

**DOI:** 10.1038/srep20170

**Published:** 2016-02-05

**Authors:** Mohamed L. Seghier, Cathy J. Price

**Affiliations:** 1 Wellcome Trust Centre for Neuroimaging, Institute of Neurology, UCL, London UK; 2 Cognitive Neuroimaging Unit, Emirates College for Advanced Education (ECAE), P.O Box 126662, Abu Dhabi, UAE

## Abstract

Functional neuroimaging studies are revealing the neural systems sustaining many sensory, motor and cognitive abilities. A proper understanding of these systems requires an appreciation of the degree to which they vary across subjects. Some sources of inter-subject variability might be easy to measure (demographics, behavioural scores, or experimental factors), while others are more difficult (cognitive strategies, learning effects, and other hidden sources). Here, we introduce a simple way of visualising whole-brain consistency and variability in brain responses across subjects using threshold-weighted voxel-based overlap maps. The output quantifies the proportion of subjects activating a particular voxel or region over a wide range of statistical thresholds. The sensitivity of our approach was assessed in 30 healthy adults performing a matching task with their dominant hand. We show how overlap maps revealed many effects that were only present in a subsample of our group; we discuss how overlap maps can provide information that may be missed or misrepresented by standard group analysis, and how this information can help users to understand their data. In particular, we emphasize that functional overlap maps can be particularly useful when it comes to explaining typical (or atypical) compensatory mechanisms used by patients following brain damage.

In multi-subject fMRI studies of brain function, effects of interest are commonly expressed in terms of significant mean group effects (i.e. a measure of central tendency). However, standard group effects do not always tell the whole story, as inferences at the group level are not always relevant (or valid) at the individual subject level^1^,^2^,^3^. For instance, Fig. 1 illustrates the not unusual situation where group effects are not even representative of the individuals that belong to that group: in (a) a significant group effect is driven by a few subjects only, in (b) a statistically significant group effect is not significant in any single subject, and in (c) a non-significant group effect reflects heterogeneity in the population with one subgroup of subjects responding differently to other subjects. Together, these examples illustrate why it would make sense to complement standard (random) group analyses with some relevant measures of consistency across subjects. Here we introduce a simple and intuitive way to visualise consistency (or variability) in individual activation maps using threshold-weighted voxel-based overlaps.

Previous analysis methods for estimating a representative group map in a multi-subject fMRI study, vary from conservative methods that down-weight the significance of an activation when there is too much variability, to more liberal methods that may reveal responses even when activation is not present in the majority of subjects; for more details see^4^,^5^,^6^,^7^,^8^. Other approaches have suggested that variability is treated as *data* rather than just noise, and that population heterogeneity can be characterised by searching for atypical subjects and clustering individuals into relatively homogenous subgroups with segregated neural systems^9^,^10^,^11^,^12^,^13^,^14^. However, the output from these methods is not always related to the individual effect in a straightforward manner, particularly for patient data when a distinction is required between an abnormal response and a noisy measurement. Indeed, in clinical fMRI, characterising atypical/abnormal patient responses requires precise knowledge of what can be considered as normal/typical in controls, which critically depends on how inter-subject variability is explained and modelled.

Beyond clinical fMRI, characterising variability in brain function is particularly useful for analyses of individual-differences^15^ that aim to look at associations between brain activations and behaviour, genetic or personality traits. Those associations may strongly depend on how effects of interest were selected. For instance, it has been shown that most brain areas that predicted the effects of practice on performance were not those that were highly activated in standard group analyses^16^. This is why others have stressed the importance of identifying ‘regions of variance’^17^, that is brain regions with the most variability across subjects, with the assumption that these regions are potentially relevant to understanding individual-differences.

One intuitive way to visualize variability across subjects at each voxel of the brain consists of generating an overlap or a frequency map over individual functional maps. Classical whole-brain overlap maps code, at each voxel, the proportion of subjects who activated that voxel at a given statistical threshold^7^,^18^,^19^,^20^,^21^. Practically, individual statistical maps are first thresholded and then summed across all subjects, so that a very consistent voxel activated in almost all subjects would appear with a high value in the generated overlap map. However, computing an overlap map necessitates the definition of an arbitrary threshold on each individual map and it can be hampered by variability in the spatial location of activated voxels across subjects^7^. Here we propose a practical solution that allows threshold-weighted overlaps to be generated at any spatial scale. We illustrate the robustness and the usefulness of such maps using real data from a group of left-handed (n = 15) and right-handed (n = 15) healthy subjects who performed a perceptual matching task on unfamiliar visual stimuli using either their left or right hand. This provided a known source of variance^11^ to illustrate the power of the functional overlap maps. We also included data from a similar task, in which participants saw the same stimuli as in the perceptual matching task but were asked to generate a consistent speech response (Say 1–2–3). For this task, there was no known source of inter-subject variability, therefore we expected the functional overlap maps to reveal consistent activation across participants.

## Materials and Methods

The protocol for this study was approved by the Ethics Committee for London Queen Square Research, and all methods and protocol were carried out in accordance with the approved guidelines. All participants provided written informed consent according to institutional guidelines.

### Subjects

30 healthy subjects (18 females, 12 males, aged 34 ± 14 years) participated in our study. According to the Edinburgh handedness questionnaire^22^, 15 were right-handed and 15 were left-handed. All subjects were native English speakers, had normal or corrected-to-normal vision, and had no history of neurological or psychiatric disorders. They were selected, chronologically, from a large cohort of neurologically normal subjects that were included in our previous studies^23^.

### Experimental design in the fMRI experiment

To illustrate the utility of the functional overlap approach, we focus on two tasks. A perceptual matching task and a speech articulation task. Both tasks were performed, in separate scanning runs, in response to the same unfamiliar stimuli. Each stimulus presented 3 different visual items, one above the central fixation point and two below (one to the left and one to the right). For the perceptual task, the participants had to indicate with a button press whether the item on the left or the right was perceptually identical to the item above. For right handed subjects (n = 15), a response indicating the selected stimulus was on the left, was made with the right index finger and a response indicating the selected stimulus was on the right was made with the right middle finger. For left handed subjects (n = 15), responses were made with the left middle finger and left index finger respectively. The choice of fingers was therefore congruent with the choice of response. For the speaking tasks, the participants were simply requested to look at the stimuli and say “1–2–3”.

There were two runs of matching and two runs of speaking. In the matching runs, the perceptual task of interest was alternated with semantic matching on familiar words and objects. In the speaking runs, the articulation task of interest was alternated with object naming and reading. Effects related to these additional tasks have been reported elsewhere and are not the focus of the current study.

Within each scanning run, there were four blocks of pictures of unfamiliar (meaningless) symbols or nonobjects. Each block lasted 18s, with 12 stimuli per block presented at a rate of three stimuli every 4.5s. There were six blocks of fixation, each lasting 14.4s. To minimize artefacts from head motion and airflow caused by the mouth opening and closing, subjects were instructed to whisper their response with minimal mouth movement. Stimulus presentation was via a video projector, a front-projection screen and a system of mirrors fastened to a head coil. Additional details about the paradigm and stimuli can be found in our previous work^23^,^24^,^25^.

### MRI acquisition

Experiments were performed on a 1.5T Siemens system (Siemens Medical Systems, Erlangen, Germany). Functional imaging consisted of an EPI GRE sequence (TR/TE/Flip = 3600 ms/50 ms/90°, FOV = 192 mm, matrix = 64 × 64, 40 axial slices, 2 mm thick with 1 mm gap). Functional scanning was always preceded by 14.4s of dummy scans to insure tissue steady-state magnetization. An anatomical scan was also acquired and later used for spatial normalization as described below. This was a 3D T1-weighted, modified equilibrium Fourier transform sequence with the following parameters: TR = 12.24 ms, TE = 3.56 ms, TI = 530 ms, FOV = 256 mm × 224 mm, acquisition matrix = 256 × 224, 1 mm slice thickness for 1 mm3 isotropic voxels.

### fMRI Data analysis

Data processing and statistical analyses were performed with the Statistical Parametric Mapping SPM5 software package (Wellcome Trust Centre for Neuroimaging, London UK, http://www.fil.ion.ucl.ac.uk/spm/). All functional volumes were spatially realigned, un-warped, normalized to the MNI space using the unified normalisation-segmentation procedure of SPM5, and smoothed with an isotropic 6-mm FWHM Gaussian kernel, with resulting voxels size of 2 × 2 × 2 mm^3^. Time-series from each voxel were high-pass filtered (1/128 Hz cut-off) to remove low-frequency noise and signal drift. The pre-processed functional volumes of each subject were then submitted to a fixed-effects analysis, using the general linear model at each voxel. Each stimulus onset was modelled as an event encoded in condition-specific ‘stick-functions’ with an inter-stimulus interval of 4.5 sec and duration of 4.32 sec per trial. Trials were grouped by blocks of 4 events (near to a configuration of a block design). The resulting stimulus functions were convolved with a canonical hemodynamic response function to form regressors for the linear model.

For each subject, we computed the contrast images for perceptual matching on unfamiliar meaningless stimuli versus saying “1,2,3” to the same stimuli. These images were then entered into a second-level analysis (i.e. random-effects analysis in SPM) so that we could identify robust and consistent activations over all our 30 subjects, with the expectation that the speech motor responses are likely to be more consistent (i.e. bilateral irrespective of handedness) compared to motor responses for finger presses (i.e. left versus right lateralization in the primary motor cortices depending on handedness).

### Voxel-based threshold-weighted overlap maps

An overlap map (OM), also called a conjunction map^20^, codes the number (i.e. percentage, proportion, frequency) of subjects that activated a given voxel, during a particular task at a given statistical threshold. It represents a practical and intuitive way to visualise consistency in activation over a given cohort of subjects^7^,^18^,^20^, and can also be considered as a measure of reliability across subjects^26^. What is attractive about an overlap map is that it does not, statistically speaking, assume homogeneity within the population. In its simplest form, it is defined as the proportion of subjects (out of *S* subjects) that activated a given voxel *v* (*v* = 1 … *V*; where *V* is the total number of voxels) at a given statistical threshold *th*:


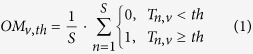


where *T*_*n,v*_ is the statistical score (e.g. a t or z value) of the *n*^*th*^ subject at the *v*^*th*^ voxel. As has been shown previously, this measure suffers from three main drawbacks: (1) its dependency on the arbitrary statistical threshold *th*, (2) anatomical variability between subjects, and (3) the spatial dependency between neighbouring voxels (e.g. voxel *v* and its nearest neighbours; see^7^).

**Figure 2:**
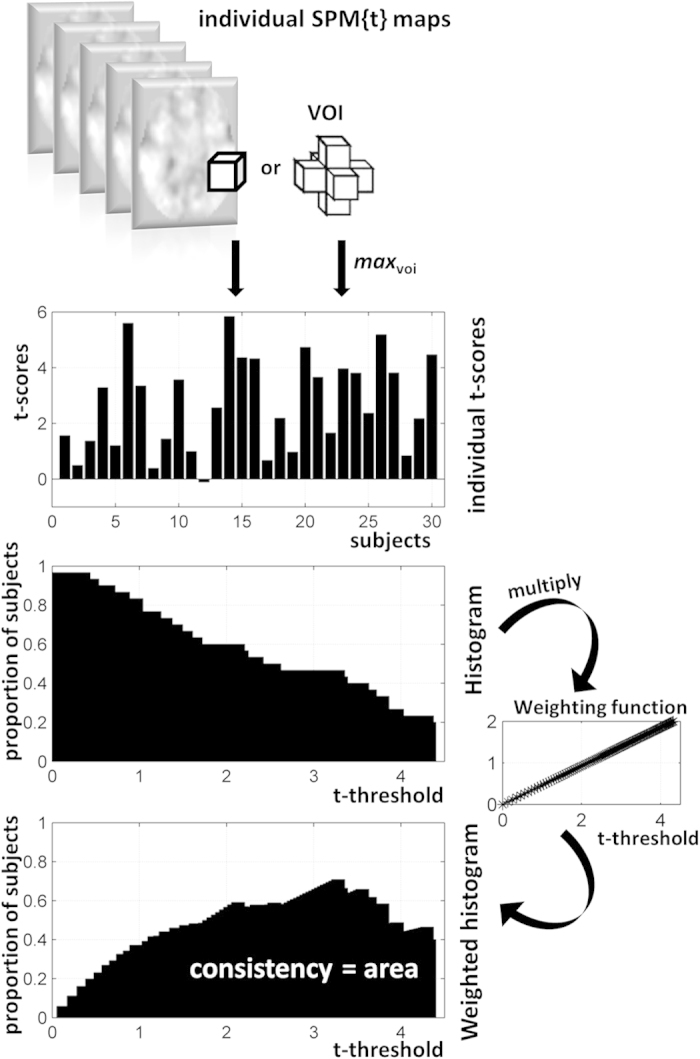
An illustration of our multi-step procedure. At each voxel, or within a VOI, (i) individual statistical values are extracted, which are (ii) transformed into a complementary cumulative histogram that is (iii) multiplied by a weighting function. The area under the curve of the weighted histogram is computed and is used to provide a measure of consistency.

Dependency on an arbitrary statistical threshold *th* is an issue that is shared by any similarity measure based on overlap between maps (see illustrations in^27^,^28^). We propose a simple and practical way that can generate threshold-weighted overlap maps at any spatial definition. We will first explain how this can be formulated in an intuitive way that can be easily implemented when using the individual SPM{t} maps as inputs. For convenience, these SPM{t} maps can be transformed into Z-maps via the corresponding p values, although this is not mandatory. Our multi-step procedure is as follows (illustrated in Fig. 2).

To define threshold-weighted overlap maps, a complementary cumulative histogram of the number of subjects against the statistical threshold *th* is assessed at each voxel *v* (i.e. the cumulative histogram is equivalent to assessing *OM*_*v,th*_ over a wider range of *th*). This idea is borrowed from the laterality index literature^29^ that has proposed ways to compute threshold-weighted laterality indices where the number of activated voxels in a given region of interest is assessed at different statistical thresholds^30^,^31^,^32^,^33^,^34^. The histogram can be assessed between a minimum *T*_*min*_ (e.g. *T*_*min*_ = 0) and a maximum *T*_*max*_ (e.g. *T*_*max*_ set at p < 0.001 uncorrected or p < 0.05 FWE-corrected). Setting a lower limit *T*_*min*_ on *th* excludes effects of non-interest (e.g. subjects showing deactivations). Conversely, setting an upper limit *T*_*max*_ on *th* minimises the effect of outliers at a given voxel (e.g. the case of a subject with extremely high t values that may dominate the histogram at high *th* if no upper limit was defined).

To reduce the information in the complementary cumulative histogram into a single useful number, we took the area under the curve of the histogram as a measure of consistency across subjects. A voxel that was activated in each subject irrespective of threshold *th* (*T*_*min*_ < *th < T*_*max*_) would display an area of 1 (expressed as a frequency or a proportion of the total number of subjects *S*). A voxel with intermediate area (<1) would either mean (1) activated in almost all subjects at lower thresholds but only survived higher thresholds (close to *T*_*max*_) in fewer subjects, or (2) consistently activated in a subgroup of subjects only irrespective of *th*. In order to assign more weight to individual effects at higher statistical thresholds, the generated histograms were subsequently multiplied by a weighting function *W*_*th*_ that monotonically increased with *th*. This weighting function, applied before estimating the area under the histogram, can be linear or nonlinear (e.g. any polynomial or exponential function). Here we used a simple linear function that increased with the threshold *th*:


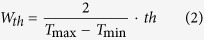


This particular shape ensured that the area of the weighted histogram fell within the range of 0 to 1 (Fig. 2). Accordingly, a threshold-weighted overlap map can be assessed at each voxel *OM*_*v*_ as following:


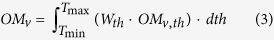


Turning now to the problem of spatial dependency between voxels and their functional/anatomical variability across subjects^35^, we need to relax the assumption that a voxel *v* is activated in each subject at exactly the same location, and allow a degree of spatial variability (or uncertainty) in the functional location of a given voxel *v* across different subjects. Such spatial variability can emerge at a larger scale than that which is typically accounted for by standard smoothing of the functional volumes during data preprocessing. Thus, our approach allows *OM*_*v*_ to be expressed at a local or regional level for tasks with known inter-subject variability in functional anatomy^21^,^36^. Functional regions can be defined by selecting the target voxel as well as its nearest neighbours (e.g. 18-connected neighbourhood); see example in^7^. Alternatively, any volume of interest (VOI) centred at each voxel *v*, with arbitrary shape and size, could be predefined and then searched for individual peaks. In this case, *OM*_*v,th*_ of Equation (3) can be substituted by:


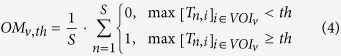


Thus, the generated histogram at a given voxel *v* summarizes the effect at that voxel plus its neighbours within the predefined VOI.

A practical advantage of working with binarised images (i.e. thresholded SPM{t} maps) is that simple morphological operations^37^ can be used to generate the overlap map. For instance, a morphological *dilation* is applied on each individual map, using the predefined VOI as a *structural element*, and then an overlap map is generated by summing the individually dilated maps. Here, to illustrate the impact of spatial variability on the generated overlap maps, we used spherical VOI with radius of 0 mm (limited to the voxel itself), 2 mm (the voxel itself plus its closest’s 6 neighbours) or 4 mm (the voxel itself plus its nearest 32 neighbours).

Using this multi-step procedure, threshold-weighted overlap maps were generated across our 30 healthy subjects for both contrasts of interest (i.e. perceptual matching versus saying 1–2–3, and the reverse contrast). As overlap maps are not designed to make statistical inferences on whether to retain or reject an effect (i.e. they can be shown in parallel with standard group SPM{t} maps), thresholding *OM*_*v*_ is optional. It does nonetheless provide insightful information of the level of consistency or variability in the population. A low value (*OM*_*v*_ towards 0) means that voxel *v* was consistently not activated in almost all subjects, a high value (*OM*_*v*_ towards 1) means that particular voxel was activated in almost all subjects irrespective of threshold *th* (*th* < *T*_*max*_), and a moderate value either means that voxel was activated in almost all subjects at lower thresholds but not at higher thresholds (close to *T*_*max*_) or consistently activated in a subgroup of subjects only irrespective of *th*. Our *OM* approach was designed to operate on the same set of voxels as in SPM. However, *OM* can also process voxels with missing data in some subjects. This can be handy for instance for datasets that do have identical brain coverage across subjects or for datasets from patients with variable lesion sites. To make statistical inferences on those voxels with missing data, users can run second-level group analyses using alternative approaches such as the GLM Flex tool (cf. http://mrtools.mgh.harvard.edu/index.php/GLM_Flex).

**Figure 3:**
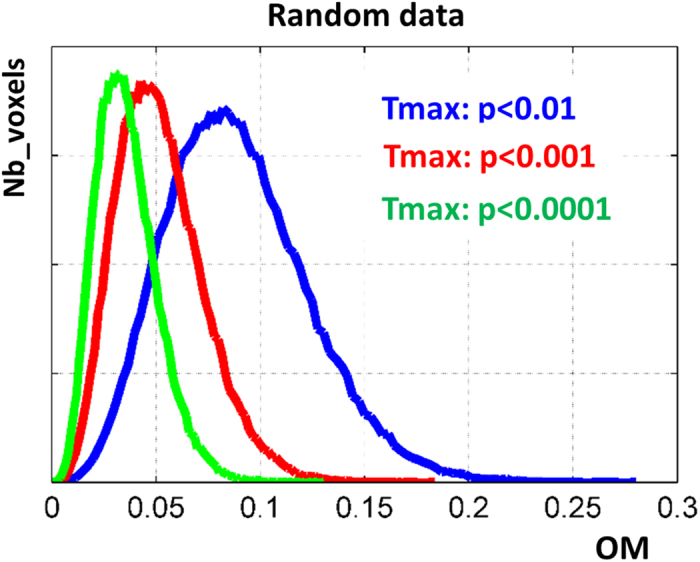
Illustrates the distribution of OM values of all voxels for random responses (blue, red and green histograms correspond to OM maps for *T*_*max*_ equivalent to p < 0.01, p < 0.001, and p < 0.0001 uncorrected respectively). The *T*_*max*_ value used during the generation of OM maps for our tasks is equivalent to p < 0.001 (red curve).

To minimise the risk of looking at *OM*_v_ values that were only due to chance, we generated *OM* maps for random responses. Practically, *S* synthetic datasets (*S* = 30) of *V* voxels (*V* = 100,000) with Gaussian noise were simulated and then the distribution of *OM*_*v*_ over all voxels were estimated at three different *T*_*max*_ thresholds (eq. to p < 0.01, p < 0.001 and p < 0.0001 uncorrected). As illustrated in Fig. 3, OM distribution was Gamma-like with OM values becoming smaller when *T*_*max*_ increased (compare red and green curves to the blue curve in Fig. 3). For instance, a threshold of 0.2 (i.e. *OM*_*v*_ > 0.2) on overlap maps with the same T_max_ value (red curve in Fig. 3), would ensure that consistent effects in our tasks across subjects cannot be due to chance only. For other datasets and parameterisations, permutation procedures can be used to set an appropriate threshold for *OM* visualisation, though this is optional.

Finally, defining a benchmark for “good consistency” depends on whether users are interested in looking at consistency or variability across subjects. These are two sides of the same coin. Obviously, an overlap of 100% (OM = 1) is good consistency, but intermediate OM values can be more difficult to interpret and motivate post hoc analyses to investigate whether they indicate meaningful variability (e.g. different subjects are using different strategies to perform the same task) or uninteresting variability (e.g. head motion artefacts correlating with task in some subjects).

## Results

**Figure 4:**
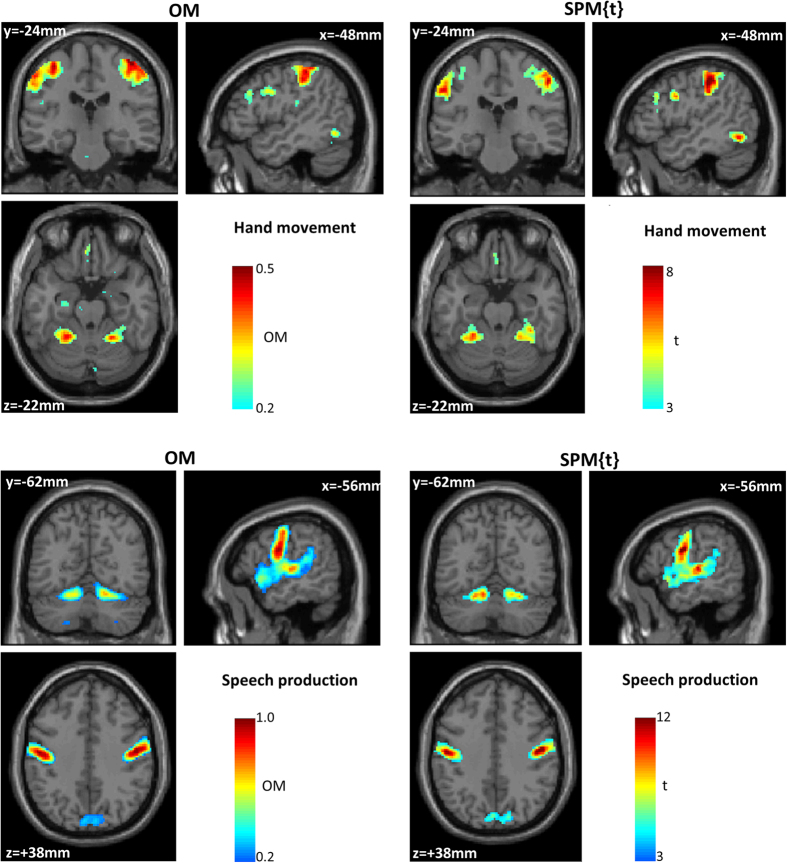
An example of threshold-weighted overlap maps (left panel) during perceptual matching (top) and saying 1–2–3 (bottom) across our 30 healthy subjects. Consistency (i.e. OM maps) is color-coded (blue = low, red = high) and thresholded arbitrarily at 0.2. The corresponding group SPM{t} maps of both tasks are shown at p < 0.001 (right panel). Note that for voxels with missing data, OM can be computed based on the available subjects that have data at those voxels.

Figure 4 illustrates a threshold-weighted overlap map for perceptual matching and saying “1–2–3”. Consistent voxels across our 30 subjects were detected in bilateral motor and somatosensory regions for both tasks with, as expected, lower consistency in the overlap map for perceptual matching because we knew a priori that subjects used either their left or right hand to do the task. The consistency of the speech motor regions was nearly 100% (Fig. 4, bottom map), suggesting that the same voxel in the speech motor regions was activated in all subjects irrespective of threshold *th* (*T*_*min*_ < *th* *<* *T*_*max*_).

**Figure 5:**
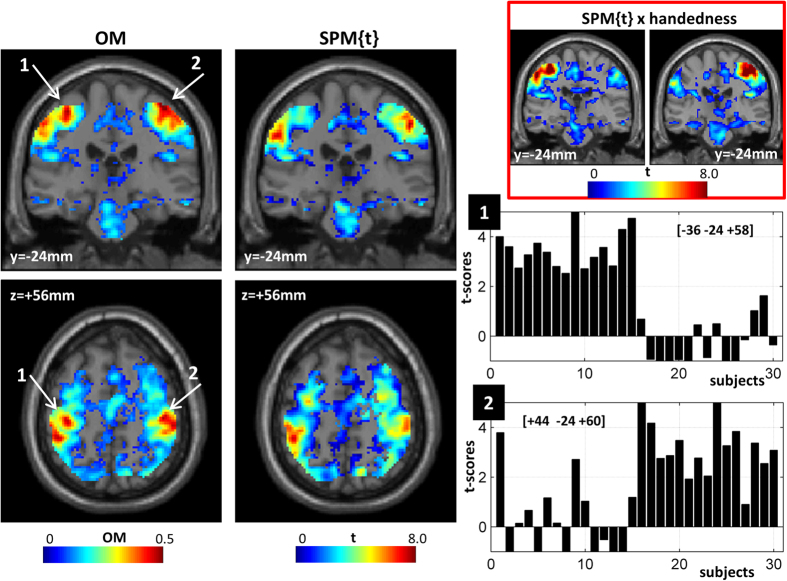
Overlap maps showing a consistent effect in the primary motor region (M1) for the hand. This was not significant (p-FWE < 0.05) in the standard group map (in both coronal and axial views). Left and right M1 are indicated with white arrows and their t-values in each subject are illustrated in the bar plots. OM = overlap map; SPM{t} = standard group analysis with random-effects as typically done in SPM. For illustration purposes, in both maps, only voxels with positive t values are shown. Typical motor activations are shown in coronal views (red box, top-right panel) using standard SPM analysis with handedness entered as a factor.

Although the SPM{t} and overlap maps are very similar, there were notable differences (Fig. 5). First, voxels in the primary motor cortex (M1) were missing from the group SPM{t} but clearly visible in the overlap map with nearly 50% subjects activating the left and right M1 regions (see bar plots of left and right M1 in Fig. 5). This was predicted a priori because we deliberately omitted to model a known source of variability (i.e. which hand the subjects were using to make a response) in order to illustrate the point. When the SPM{t} analysis is repeated with handedness as a factor, the left and right primary motor activations are uncovered (see coronal views, top-right panel in Fig. 5). The point is that inconsistency in the overlap maps can indicate where the standard GLM approach might be improved by modelling known sources of between-subject variance. It is not necessarily indicating a problem with the GLM approach per se.

**Figure 6:**
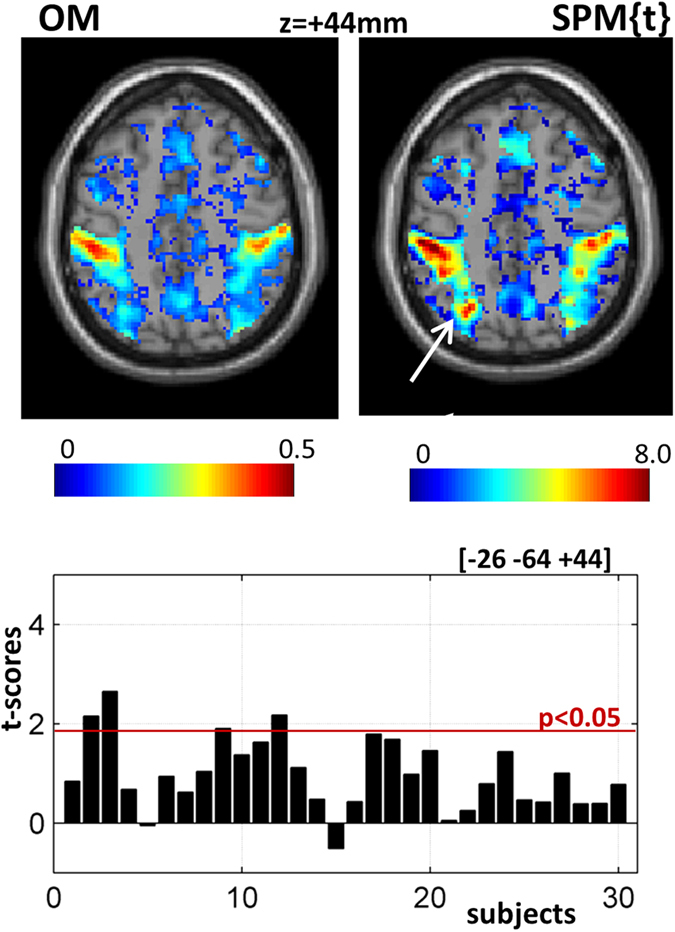
Illustrates a strong effect in the parietal lobule from the standard group analysis that was not highly consistent (i.e. activated) in each individual subject.

On the other hand, a significant cluster in the superior parietal lobule (Z-score = 5.5, p < 0.05 FWE-corrected) in the group SPM{t} map did not show up with high consistency in the overlap map (Fig. 6), because it was weakly activated or absent in the majority of subjects. It reached significance in the SPM{t} map because variance was very low with positive activation in 28/30 subjects, even though this only surpassed a threshold of p < 0.05 uncorrected in 4 subjects (cf. bar plot in Fig. 6).

**Figure 7:**
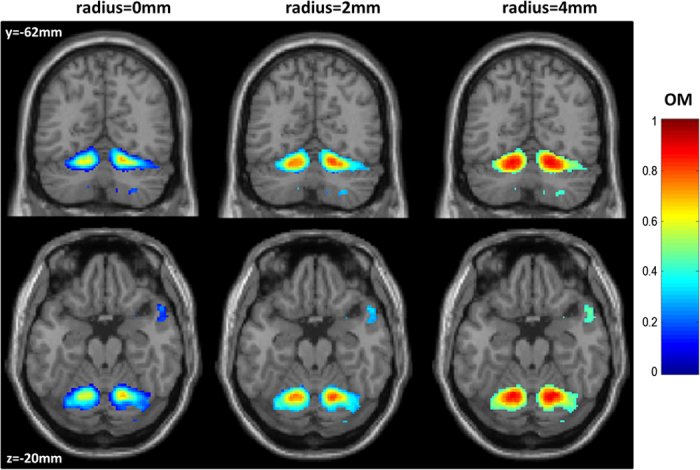
Shows consistency in the cerebellar regions for saying 1–2–3 using different VOIs: the voxel itself (left), the voxel itself plus its neighbours within a 2 mm-radius sphere (middle), or the voxel itself plus its neighbours within a 4 mm-radius sphere (right).

Last but not least, the consistency across subjects and maps is improved by taking into account variability in the location of activated voxels across subjects (by including neighbouring voxels). This is illustrated with the cerebellar regions (Fig. 7) that are known to be highly variable even after spatial normalization with SPM5^38^. Using a small VOI (a 2 mm-radius sphere), it was possible to account for up to one voxel mismatch across subjects and thus substantially increase the consistency of the cerebellar activations associated with saying “1–2–3” (Fig. 7).

## Discussion

In this study, we demonstrate a new flexible way of generating whole-brain overlap maps of functional activations across subjects. These functional overlap maps complement standard group analyses by indicating how consistently a given effect occurs across subjects. This is particularly useful when it comes to understanding inter-subject differences in relation to the conclusions that are valid for the group^39^. Below, we discuss the advantages of the functional overlap maps we are proposing relative to standard GLM techniques for investigating inter-subject variability, how these overlap maps differ from other overlap or conjunction maps, how the degree of consistency can be interpreted, other uses of overlap maps and methodological issues.

### How overlap maps can supplement results from GLM analyses

Many studies have shown that reliance on group maps alone may be incomplete^1^,^2^,^3^,^16^, which stresses the need for complementary information about individual effects and their consistency. The advantages of using functional overlap maps in addition to standard GLM techniques for investigating inter-subject variability are as follows: First, our functional overlap maps provide a quick and easy image of the whole brain response, during any given condition, that indicates where activations at a given voxel/region in the GLM have emerged from (a) the whole sample (e.g. 100% of subjects); (b) subgroups (e.g. 50% of subjects) and (c) atypical participants (e.g. <10% of subjects). Second, these quick and easy to read maps can motivate informative post hoc analyses of inter-subject variability that might be neglected in studies based on GLM analyses only. Third, they can facilitate studies of inter-subject variability by guiding attention to the regions that are most informative, some of which would not easily be detected in standard GLM analyses; for example, if a region was activated by half the subjects and deactivated by the other half (resulting in zero mean activation).

Fourth, knowing the full spectrum of consistency and variability across subjects is important for inferring the likely causes of inter-subject variability because if post hoc analyses only investigate measureable known factors (e.g. behaviour, demographics, and experimental factors), then other “hidden” sources of variability (e.g. genetic, individual preferences, and educational factors) could be missed by standard GLM analyses^40^,^41^,^42^. Conversely, by knowing that all the regions identified in a group GLM were consistently activated across subjects, there can be greater confidence when excluding the contribution of hidden factors to the functional architecture of the sample (although these factors may still affect the degree to which each region is activated).

Fifth, when variability is caused by a mixture of measurable and hidden factors, knowing which regions are most variably activated across subjects can increase sensitivity to measurable effects of variability. For example, if one subgroup of participants activate a region, and another does not and the subgroups do not differ in measurable ways (e.g. behaviour, demographics), further investigation of measured sources of variability can be focused within each subgroup^10^ (i.e. after controlling for other major but hidden sources of variability).

Sixth, all the above factors can help to interpret what is normal and abnormal in the activation pattern seen in a patient. This is particularly useful when a patient has damaged/lost an area of the brain that is significantly activated in a GLM analysis but shows no significant difference relative to normal activation despite being able to perform the task. Functional overlap maps can be used to predict this potentially surprising result *a-priori*, by showing that the damaged area is not consistently used in normal subjects despite the high group activation. The normal control group can then be tailored to those who don’t typically use the damaged area (i.e. those that are most like the patient). We can then ask whether the patient maintains or recovers the ability to perform the task using a neural system that is also used by the selected control group but not the remaining controls.

Likewise, significantly greater activation in a patient relative to a group of healthy controls does not necessarily mean that the patient had more activation than each of the controls. It only means that the patient response was higher than the average control response. Overlap maps of activation over all healthy subjects allow the user to visualise how atypical patient responses fit with normal consistency and variability, which is useful for motivating richer, more accurate mechanistic explanations of clinically relevant effects. More specifically, an explanation of an abnormal effect might indicate (i) atypicality, i.e. when not activated in any individual healthy subject, (ii) use of one of several possible normal neural systems that can each sustain the same task, i.e. when activated in a subset of controls only, or (iii) enhanced reliance on a neural system that is used by all controls, i.e. when activated in almost all controls but at a lower amplitude than the patient, perhaps because of less effort.

Finally, overlap maps can be particularly useful for assessing aggregate effects in heterogeneous clinical populations. In fact, they might be the only meaningful way to combine heterogeneous patient maps^43^, when patients differ markedly with respect to their lesions and recovery trajectories.

### Other uses of functional overlap maps

Our method also provides a useful tool to assess the detectability power of a given fMRI paradigm at the individual subject level, which is known to vary with both task and region^7^,^8^,^44^,^45^. This can be achieved by generating overlap maps for each contrast of interest at any given spatial (regional) level. Another application concerns the widely used practice of selecting regions of interest. For instance, functional connectivity studies typically need to limit data to those from a particular set of regions (nodes). The coordinates of such regions are commonly defined from the group analysis; however, this does not necessarily guarantee that the same region is activated in each subject, which sometimes requires that the subjects with missing values in regions of interest are excluded. For example, although a left superior parietal cluster was significantly activated in our group analysis (Fig. 6), only 4 out of 30 subjects activated that cluster at a liberal threshold of p < 0.05 uncorrected. This is why that cluster appeared with very low consistency in the overlap map (Fig. 6). The apriori measure of subject consistency that our overlap maps provide is therefore particularly useful for region selection in connectivity analyses.

The complementary information provided by overlap maps can also indicate when users need to consider better or more useful GLM models. This information can be generated at the voxel/regional level, where there is least homogeneity in the group/population. In the example we provide, we deliberately omitted a known source of variability (i.e. hand response) from our group analysis to show how this would be detected in the overlap maps but not the SPM{t} maps. After confirming that variability in the overlap map corresponded to whether the participants used their left or right hand, we created a better group analysis that modelled inter-subject variability in hand responses. This approach to improving the group analysis on the basis of viewing an overlap map could be used in other contexts where the degree of inter-subject variability is unknown. It could, for instance, involve including a range of possible explanatory variables (i.e. sources of variance) into the group analysis models, testing for normality, using non-parametric statistics, dealing with outliers, using robust statistics or modelling the group as a mixture of subpopulations^8^,^9^,^10^,^11^,^12^,^13^,^14^,^46^,^47^,^48^,^49^.

### Comparison with other methods and methodological issues

The difference between our maps and those presented previously^7^,^18^,^19^,^20^,^21^ is that they show consistency and variability, within the same whole brain image, taking into account a wide range of statistical thresholds. The advantage of considering multiple statistical thresholds is that we avoid the application of arbitrary thresholds to individual maps that might vary in both the spatial location and hemodynamic response to effects of interest^7^.

Unlike previous approaches, our overlap maps can also be shown at any spatial scale (from the voxel to the regional level) and do not require any assumption about the normality or the homogeneity of the population. It is easy to implement as it can operate directly on the already computed individual SPM{t} maps. Compared to variance maps^17^, overlap maps do not depend on estimates of within-subject variance. The flexible scheme we propose here can also be applied to other measures of brain activation, for instance on the basis of the signal amplitude (i.e. effect size) in individual subjects^50^,^51^,^52^ rather than their statistical scores.

Now we turn to our multi-step procedure (Fig. 2), to consider the influence of *T*_*max*_, the number of subjects *S*, the shape of the weighting function *W*_*th*_ (Equation (2)), the size of the VOI, and the meaning of moderate *OM* values.

### The influence of *T*_*max*_

Our approach integrated the areas of the histograms (i.e. number of subjects against threshold, as in Fig. 2) over a wider range of thresholds instead of searching for an optimal threshold that is subject-specific (e.g. as is the case for instance in test-retest fMRI protocols^28^,^53^). The definition of an upper limit on the range of statistical thresholds makes our approach robust to outliers. It is obvious to see that consistency values in the overlap maps would decrease with *T*_*max*_ because the effective number of subjects who activated the same voxels at very high thresholds is likely to decrease (also illustrated in Fig. 3 using synthetic data). The overlap maps generated at different *T*_*max*_ values are expected to be strongly correlated because, by construction (Fig. 2), subjects who contribute to OM_v_ at a higher *T*_*max*_ value are also contributing to OM_v_ at a lower *T*_*max*_ value. For example, when calculating the voxel-wise correlation of OM_v_ values from the perceptual matching contrast at four different *T*_*max*_ values (equivalent to p < 0.01, p < 0.001, p < 0.0001, and p < 0.00001 uncorrected), all pairwise correlations were larger than 0.9. When comparing overlap maps between different groups or tasks, *T*_*max*_ must be held constant. Here we recommend the use of a *T*_*max*_ equivalent to p < 0.001 (uncorrected).

### The influence of subject numbers

Our approach can be applied to any sample, though the number of subjects *S* will define how ‘smooth’ the cumulative histogram is, given that discrete quantities are manipulated during the assessment of *OM*_*v*_. Specifically, for a given sample size *S*, the difference between two bins (Fig. 2) is always a multiple of 1/S and the possible number of discrete levels (i.e. *OM*_*v,th*_ between 0 and 1) in the histogram is less than or equal to S + 1. Thus, a smaller *S* resulting in a coarser histogram. Critically, this ‘digitisation’ does not hamper the assessment of the area under the curve of the histogram (cf. Equation (3)), given that *OM*_*v*_ is identifiable even for voxels with few activating subjects (e.g. voxels in blue in Fig. 5 that mimic the case of samples with small subject numbers.

### The influence of the weighting function

**Figure 8:**
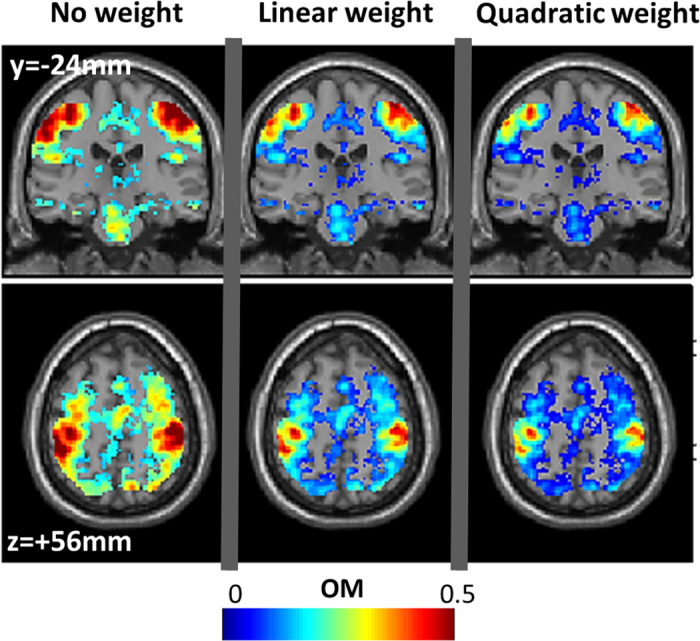
illustrates the impact of the weighting function *W*_*th*_on consistency values in the overlap map. The same coronal and axial views as in Fig. 5 are shown. Three polynomial functions were applied: Left panel: *W*_*th*_ = 1; middle panel: *W*_*th*_ = 2*t/T_max_; left panel: *W*_*th*_ = 3*(t/T_max_)^2^. During the assessment of the three overlap maps, the following parameters were held constant: *T*_*max*_ equivalent to p < 0.001 uncorrected, *T*_*min*_ = 0, and no spatial filtering (VOI limited to the voxel itself).

The weighting function *W*_*th*_ can be of any monotonic form^32^,^33^, but it is also valid to compute the same consistency values without weighting. The rationale here would be to boost the consistent effects that are significant in many individual subjects at higher thresholds. Likewise, a weighting function *W*_*th*_ would down-weight the impact of individual effects at lower thresholds, thus, as those effects are the dominant ones (i.e. the cumulative histogram decreases with threshold *th*), stronger nonlinear weighting functions would yield smaller values in the overlap maps (see illustration in Fig. 8).

### Adjusting the volume of interest (VOI)

By applying a nonlinear spatial filtering (Equation (4)), it is possible to predefine a VOI of any shape to limit the extent of the spatial dependency between neighbouring voxels. We recommend the use of small spherical volumes of interest (e.g. the 6 closest neighbours to every voxel that touches one of their faces), although users can select larger VOI if the peaks of activated regions are expected to be particularly variable across subjects. As expected (Fig. 7), a larger VOI yields smoother overlap maps.

### Interpreting moderate values in the overlap maps

As mentioned above, standard overlap maps that compute the proportion *N* out of *S* subjects at a given statistical threshold *th* are easy to interpret but are critically dependent on *th*^7^,^18^,^19^,^20^. To deal with this threshold dependency, our new approach collapses two dimensions (number of subjects and threshold) into one measure that we refer to as a weighted-threshold OM value (cf. Fig. 2). The compromise is that the interpretation of intermediate OM values becomes slightly ambiguous. For example, if *N* out of *S* subjects activated a particular voxel/region irrespective of threshold *th*, then it is straightforward to show that OM is equal to the ratio 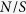 (noted Scenario 1). However, by construction, if all subjects *S* activated that voxel up to a particular threshold *T*_*p*_ (with T_p_ < T_max_), then it is straightforward to show that OM would also show the same value 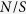 if *T*_*p*_ is equal to 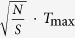 (noted Scenario 2). This illustrates that the same intermediate OM value can result from two different scenarios. To improve the interpretability of intermediate OM values, we propose two practical solutions. The first one simply proceeds by regenerating an overlap map at a lower *T*_*max*_ threshold with the expectation that OM values for voxels of Scenario 1 will not change whereas OM values for voxels of Scenario 2 would increase. The second way that we recommend here is to read the overlap map in parallel with the SPM{t} group map, with the expectation that voxels of Scenario 1 are more likely to have low t scores (e.g. mixture of subgroups yielding high between-subject variance) whereas voxels of Scenario 2 are more likely to have high t scores (e.g. consistent small individual effects yielding low between-subject variance). In all cases, when it comes to reject or retain an effect, we recommend that all statistical decisions to be made on the basis of the standard SPM{t} maps.

## Conclusion

In summary, threshold-weighted overlap maps are easy to generate and can provide useful complementary information about individual effects. They supplement and facilitate post hoc analyses/re-analyses of GLM results by informing the user about the (potential) existence of other sources of heterogeneity in the data (e.g. unknown sources of variance) that might not be explicitly taken into account when assuming homogeneity/normality of the group data.

The interpretation of data from many scenarios would benefit from threshold-weighted overlap maps. For example, data from tasks that are expected to involve different cognitive strategies and hence different supporting neuronal systems^40^,^54^, or when there are individual differences in learning or subjective judgment^55^,^56^, or when mapping functional responses that are expected to be spatially variable across individuals^21^,^57^. In our future work, we are planning to generate threshold-weighted overlap maps, across hundreds of healthy subjects, for many different language and sensory functions^58^, which can serve as an fMRI normative database for clinical applications.

## Additional Information

**How to cite this article**: Seghier, M. L. and Price, C. J. Visualising inter-subject variability in fMRI using threshold-weighted overlap maps. *Sci. Rep.* **6**, 20170; doi: 10.1038/srep20170 (2016).

## Figures and Tables

**Figure 1:**
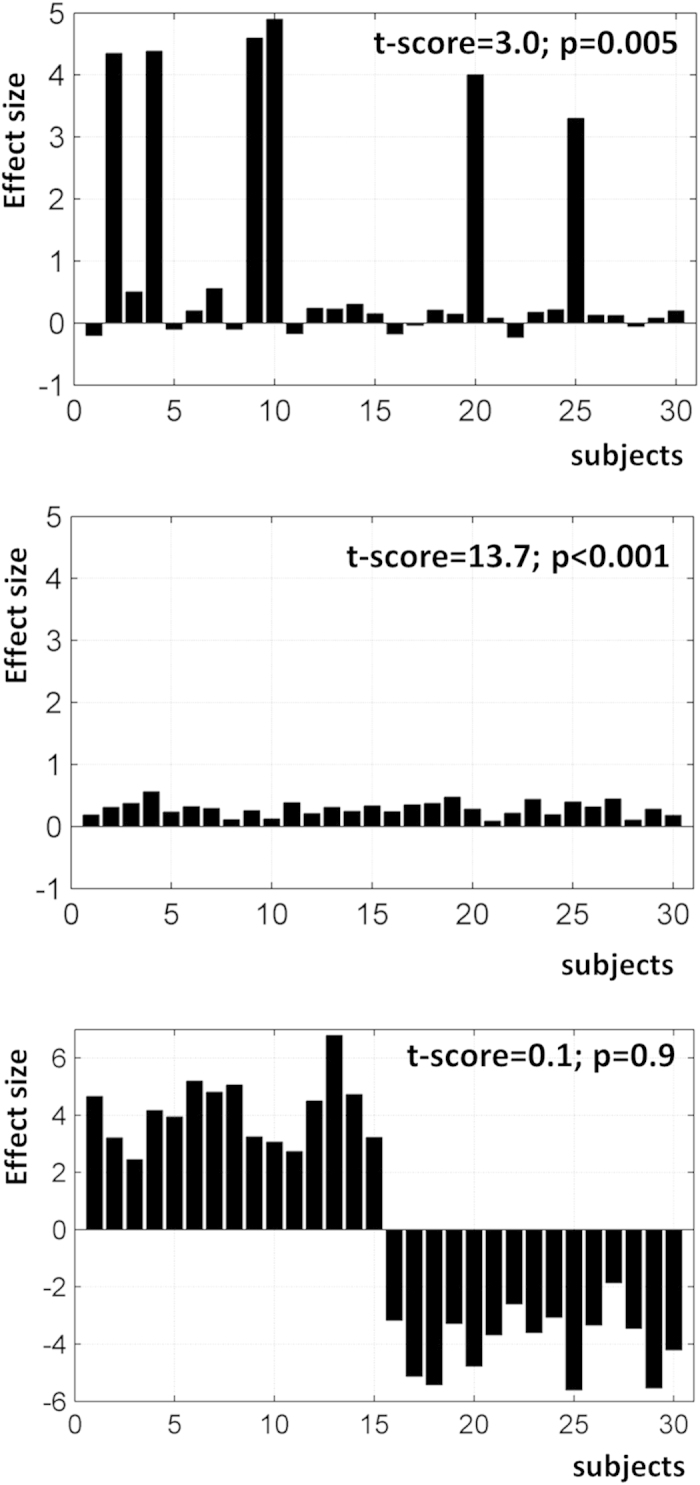
Illustrates a hypothetical example (synthetic data) of three group effects across 30 subjects where overlap maps can be very handy. (top) A significant group effect driven by a few subjects with atypically strong activation; (middle) a significant group effect due to consistent but small effects in each individual; (bottom) a non-significant group effect caused by huge heterogeneity as half of the subjects responded completely differently to the other half.
